# Elbow spasticity during passive stretch-reflex: clinical evaluation using a wearable sensor system

**DOI:** 10.1186/1743-0003-10-61

**Published:** 2013-06-19

**Authors:** Chris A McGibbon, Andrew Sexton, Melony Jones, Colleen O’Connell

**Affiliations:** 1 Institute of Biomedical Engineering, University of New Brunswick, 25 Dineen Dr., PO Box 4400, Fredericton, NB E3B 5A3, Canada; 2 Faculty of Kinesiology, University of New Brunswick, Fredericton, NB Canada; 3 Stan Cassidy Centre for Rehabilitation, Fredericton, NB Canada

**Keywords:** Spasticity, Upper motor neuron syndrome, Modified Ashworth scale, Elbow muscle tone

## Abstract

**Background:**

Spasticity is a prevalent chronic condition among persons with upper motor neuron syndrome that significantly impacts function and can be costly to treat. Clinical assessment is most often performed with passive stretch-reflex tests and graded on a scale, such as the Modified Ashworth Scale (MAS). However, these scales are limited in sensitivity and are highly subjective. This paper shows that a simple wearable sensor system (angle sensor and 2-channel EMG) worn during a stretch-reflex assessment can be used to more objectively quantify spasticity in a clinical setting.

**Methods:**

A wearable sensor system consisting of a fibre-optic goniometer and 2-channel electromyography (EMG) was used to capture data during administration of the passive stretch-reflex test for elbow flexor and extensor spasticity. A kinematic model of unrestricted passive joint motion was used to extract metrics from the kinematic and EMG data to represent the intensity of the involuntary reflex. Relationships between the biometric results and clinical measures (MAS, isometric muscle strength and passive range of motion) were explored.

**Results:**

Preliminary results based on nine patients with varying degrees of flexor and extensor spasticity showed that kinematic and EMG derived metrics were strongly correlated with one another, were correlated positively (and significantly) with clinical MAS, and negatively correlated (though mostly non-significant) with isometric muscle strength.

**Conclusions:**

We conclude that a wearable sensor system used in conjunction with a simple kinematic model can capture clinically relevant features of elbow spasticity during stretch-reflex testing in a clinical environment.

## Background

### Motivation and problem statement

Persons with upper motor neuron syndrome (UMNS) due to brain or spinal cord lesion often present with muscle spasticity which interferes with basic motor tasks required for self-care and independence, and is a major contributor to mobility impairment and disability in this population [1]. Spasticity is characterized by an involuntary velocity-dependent stretch reflex with exaggerated tendon jerks (the tonic and phasic reflex mechanisms, respectively), that causes the stretching muscle to activate inappropriately during passive and active movements [2], and an abnormal increase in muscle tension that increases resistance to passive motion [3]. Associated symptoms include muscle weakness and reduced range of motion [4].

Whether treatments are activity based (such as stretching, bracing), pharmacologic (such as oral and intrathecal antispasticity agents or chemodenervation), physical (such as bracing), or surgical (such as dorsal rhizotomies), quantifying spasticity is essential for optimal management [5]. However, tools available to clinicians for objectively quantifying spasticity are limited. Clinical case load management constraints and general lack of resources has firmly entrenched a “low-tech” approach to neurological assessment. As such, therapists still rely on subjective rating scales for assessing spasticity, such as the Modified Ashworth [6] and Tardieu scales [7]. This holds true for most outcomes measures used in clinical trials related to spasticity treatment [8].

Technologies for accurate and objective assessment of these indicators do exist [9-12] but are limited to laboratory-based research and are not suitable or accessible for routine clinical use. Despite the appeal of incorporating minimally invasive sensor technology in the clinic, such as an instrumented version of the stretch-reflex test, only a few researchers have explored this topic [13,14].

It is also still unclear how to interpret instrumented or sensor-based measures of spasticity and their relationship to clinical rating scales [15]. Studies range from showing no correlation between neuro-biomechanical measurement and clinical scales such as the MAS [11,12,16,17] to showing significant correlation between muscle EMG responses and MAS [18-21]. However, much of the prior work in this area is laboratory-based, using mechanical devices such as torque motors and robots to move the limb through its range of motion or to apply controlled perturbations to the limb, and therefore may not explain aspects of spasticity that are being measured during manual assessment (such as passive stretch-reflex performed by a therapist).

### Proposed solution to stated problem

In order to explore this issue a wearable sensor system (the “BioTone”) was developed for capturing elbow joint flexion/extension kinematics and flexor and extensor muscle electromyography (EMG) during a clinical stretch-reflex test, as used when administering the MAS in a clinical setting. Unlike lab-based mechanical or robotic devices that provide controlled smooth or perturbed movement profiles, our clinically-based system recorded the disturbed kinematic profile caused by patient’s muscle activation during the passive stretch motion induced by the therapist. Muscle EMG was measured simultaneously for flexor and extensor muscles. A model was derived (described below) based on trial-specific passive kinematics to detect the onset and intensity of the spastic stretch-reflex. Portable electronic tools were also used to acquire objective strength and passive range of motion measures. The therapist administering the tests also provided a clinical MAS score for both elbow extensor and flexor spasticity, which enabled us to explore the relationship between the biomechanical and electrophysiology responses and with those assessed using a routine clinical assessment tool.

### Research purpose and objectives

The purpose of this research was to establish the construct validity of using a wearable sensor system for elbow flexor and extensor spasticity assessment. We had two objectives: 1) to assess the kinematic and EMG responses from the BioTone system during stretch-reflex testing and develop a framework for extracting metrics relevant to spasticity assessment; and 2) to examine the relationship between these sensor-based metrics and clinical MAS score, as well as strength and passive range of motion.

## Methods

### Participants

At the time this preliminary analysis, 25 patients were recruited for assessment of spasticity using a wearable sensor system called the “BioTone”, developed by the Institute of Biomedical Engineering at the University of New Brunswick (IBME, Fredericton NB, Canada). All participants were recruited through the Stan Cassidy Centre for Rehabilitation (SCCR, Fredericton, NB), the provincial tertiary neurorehabilitation hosptial. Included were adult patients with acquired brain injury (ABI), cerebral palsy (CP), multiple sclerosis (MS) and spinal cord injury (SCI). Of the 25 patients included in the study thus far, 12 were being treated for upper-extremity muscle spasticity. Three were excluded due to technical difficulties during data collection. Therefore, data for nine participants were included in the present analysis. Demographic and diagnostic data for the study sample are shown in Table 1. The research was reviewed and approved by both the University and Regional Health Authority Research Ethics Boards, and all participants provided informed signed consent prior to data collection.

**Table 1:** Demographic and diagnostic data for study participants

							**Strength (N)**	
**ID**	**Gender**	**Age**	**BW (kg)**	**D**_X_*	**Side**	**PROM (**^**o**^**)**	**Flexor**	**Extensor**
1	M	41	76.0	ABI	R	123.31	3.94	19.58
2	M	73	77.8	MS	L	131.23	35.19	40.15
3	F	39	74.5	CP	L	128.74	17.25	20.04
4	F	67	76.8	MS	L	112.75	11.30	6.16
5	F	62	60.6	ABI	L	114.66	3.72	13.50
6	F	46	98.7	ABI	R	104.40	17.47	33.89
7	M	33	84.5	ABI	L	75.08	3.92	20.67
8	M	28	73.9	ABI	L	95.26	47.14	50.13
9	M	40	59.0	SCI	L	121.70	42.45	68.80

Data shown are for most affected side (subjects 3 and 6 were being treated for bilateral elbow spasticity).

* Diagnostic categories were *ABI* acquired brain injury, strokes; *MS* multiple sclerosis, *CP* cerebral palsy, *SCI* spinal cord injury.

### Experimental protocol

Participants were evaluated in a clinical setting (SCCR) by a licensed physiotherapist. Therapists participating in the study were already qualified or trained (by M.J.) to administer the MAS assessment, and were subsequently trained to operate the BioTone hardware and software during administration of the stretch-reflex test (by A.S.). All measurements were acquired by sensor devices worn by the participant during the examination by the therapist. For this study, only data from the affected (or most affected) limb was measured.

#### BioTone system

**Figure 1:**
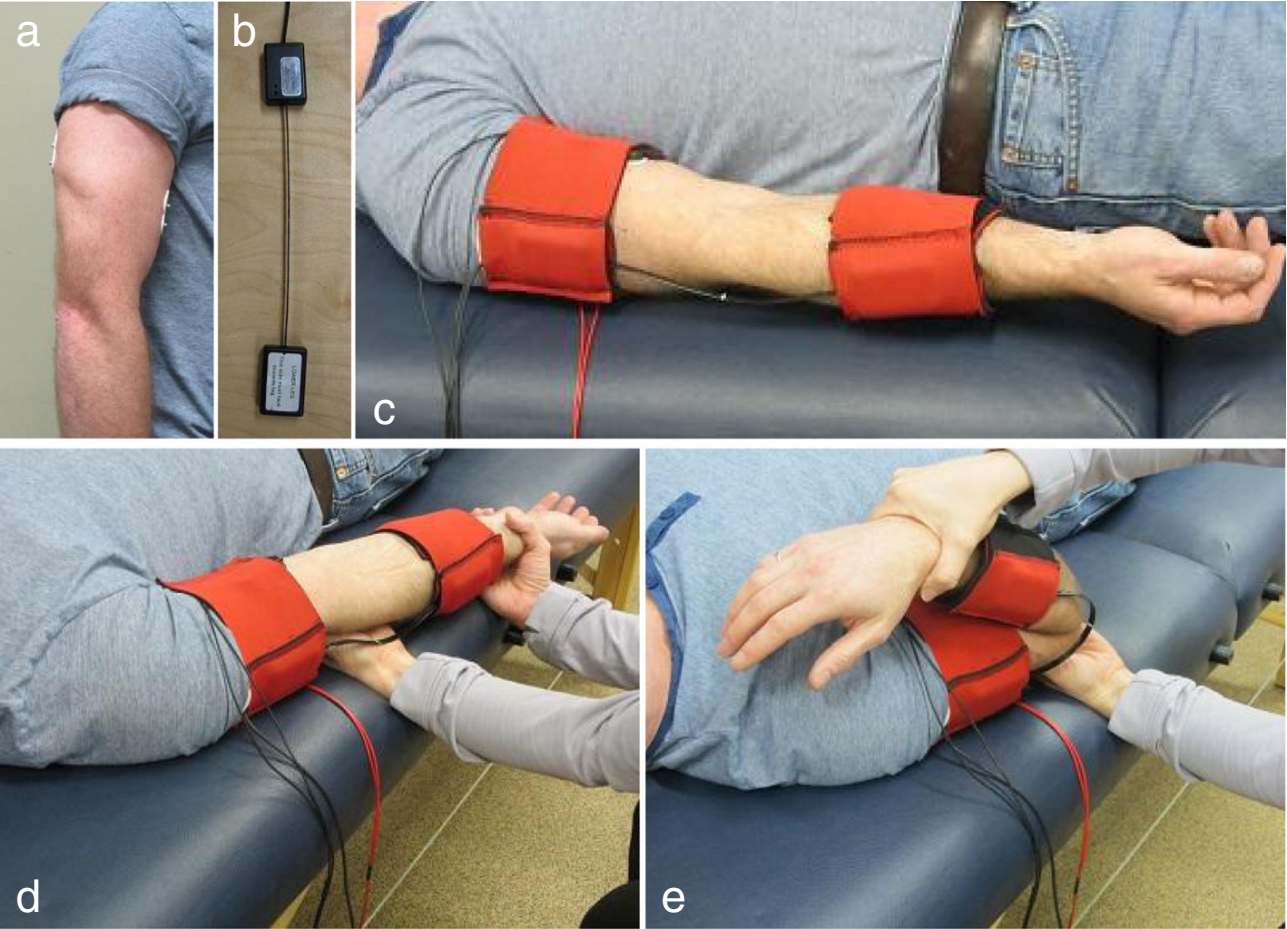
**BioTone tools used in the evaluation of spastic hypertonia.** Top: **a**) Electrode placement for triceps and biceps with 2-channel EMG system; **b**) The ShapeSensor™ fibre-optic goniometer; and **c**) The arm instrumented with a wearable sensor system. Bottom: Stretch-reflex test being performed by a therapist with: **d**) limb in extension; and **e**) limb in flexion.

BioTone hardware consists of a 2-channel EMG system (custom designed by UNB, that uses DuoTrode Ag-AgCl electrodes, Figure 1a), and a single degree of freedom fibre-optic goniometer (ShapeSensor™, Measurand Inc., Fredericton, NB, Figure 1b) mounted as shown Figure 1c). Sensors connect to an analog interface (BioSI™, custom design by UNB, not shown) that controls sampling and sends data to the laptop computer for storage, processing and real-time graphic display. The BioTone software guides the therapist through the testing protocol (including order of trials) and records all data during the therapist’s examination of participants. It also provides real-time display of sensor data. For this study the therapist only used the EMG trace for gauging when the participant’s muscles were quiet enough to commence a trial, and the angle sensor trace to ensure consistency in testing.

#### Stretch-reflex testing

To evaluate muscle spasticity, participants underwent passive stretch-reflex testing of the elbow in flexion and extension according to methods described for administering the MAS in clinical practice, originally described by Ashworth [22] (and scoring method modified by Bohannon [6]). The therapist then recorded a MAS score for elbow flexion and extension based on subjective ratings shown in Table 2. This was performed prior to collecting data so the therapist could not be influenced by sensor responses when arriving at a MAS score.

**Table 2:** **Modified ashworth scale description and the scoring method used for data analysis**[7]

**MAS score**	**Score used in analysis**	**Descriptor**
0	0	No increase in muscle tone
1	1	Slight increase in muscle tone, manifested by a catch and release, or by minimal resistance at the end of the range of motion when the affected part(s) is moved in flexion or extension
1+	2	Slight increase in muscle tone, manifested by a catch, followed by minimal resistance throughout the remainder (less than half) of the range of movement (ROM)
2	3	More marked increase in muscle tone through most of ROM, but affected part(s) easily moved
3	4	Considerable increase in muscle tone, passive movement difficult
4	5	Affected part(s) rigid in flexion and extension

During data collection, the fibre-optic goniometer and 2-channel surface EMG electrodes (biceps bracii and triceps bracii) were worn by the participant for recording elbow kinematics and muscle activity during the stretch-reflex test. Devices were mounted using a custom designed cuff system that allowed quick donning and doffing by the therapist, as illustrated in Figure 1. Kinematic and EMG data were collected at 1000 Hz for eight trials, as follows. First, a single slow (~10-20 deg/s) flexion and extension trial (throughout the passive range) was performed, followed by a series of fast (~120-140 deg/s) flexion and extension trials (throughout the same passive range). A short rest period was used between tests to allow muscles to relax, which was monitored from the real-time display on the laptop computer. Fast flexion and extension trials were each performed three times. Testing positions in extension and flexion are shown in Figure 1d and 1e, respectively.

Because the MAS scale (0–4) includes the inconvenient category of 1+, the MAS scores were re-assigned to a 0–5 scale. Description of the MAS score definitions from Bohannon and Smith [6] and our conversion scores for analysis are summarized in Table 2.

#### Strength and ROM testing

Elbow flexion and extension passive (PROM) range of motion were collected using the elbow goniometer system. The therapist moved the participant’s limb slowly through its available range, a total of three times, and for each trial the minimum angle (extension) and maximum angle (flexion) were recorded.

Elbow flexor and extensor isometric strength were measured using a custom designed wearable dynamometer [23] for quantifying isometric elbow muscle torque (at 90 deg) in flexion or extension. The dynamometer was placed on the participant’s arm, secured in place, and then used to record three trials at maximal effort for both elbow extensor and elbow flexor muscles.

### Data processing

Our first objective was to develop a framework for extracting sensor-based metrics that reflect spastic response during stretch-reflex testing. Because the kinematic profiles captured during a spastic response reflect both the therapist driving the participant’s forearm and the participant’s involuntary muscle responses, we first required a method for modeling the therapist’s intended motion profile. We approached this from the standpoint that resistance-free passive motion would result in the participant’s forearm motion profile being identical to the therapist’s intended movement profile, but that resistance encountered would cause a departure from the therapist’s intended (or reference) profile. The degree of kinematic departure from this reference profile should correspond to the intensity of EMG response.

The following sections describe a model of passive joint motion that represents the therapist’s intended motion profile and therefore enables the participant’s actual movement and EMG profiles to be compared to a reference profile.

#### Kinematic model of passive elbow motion

**Figure 2:**
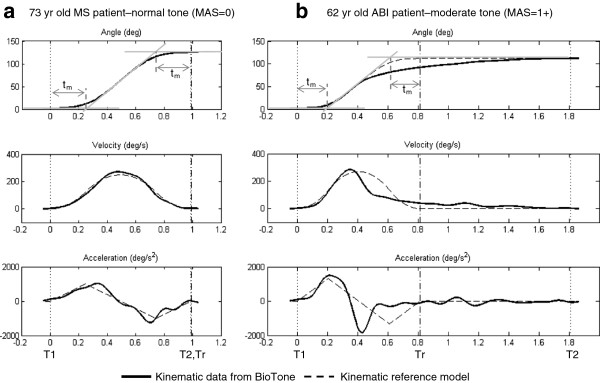
**Derivation of the kinematic reference profile for two representative patients.** BioTone data for: **a**) patient with normal flexor tone (MAS = 0), and **b**) patient with moderate tone (MAS = 1+). Displacement curves are shown in the top row. Gray construction lines illustrate the analytical approach to defining a time interval (T1-Tr) to represent the intended kinematic (reference) profile by the therapist. Within the interval T1-Tr an acceleration profile was constructed to represent a constant jerk curve (bottom row). Actual and reference kinematic profiles are indicated by bold and dashed lines, respectively. T2 represents the end of the motion.

The kinematic model was based on a smooth (constant jerk) profile, as this appeared to explain quite well the observed passive motion induced by the therapist. A custom interactive program was written in MatLab (MathWorks Inc. Natick, MA) to do this processing off-line. Angle data collected from the BioTone were first low-pass filtered at 6Hz (zero-lag, 4^th^ order Butterworth). Then, the start time (T1) and end time (T2) of elbow motion were selected to establish the upper and lower limits of motion. Second, the maximal slope was determined from the initial movement portion (prior to spastic disturbance), and then used to define the time (t_m_) between T1 and intersection of the slope with a projected line representing the minimum angle. The slope intersection with the projected line from the maximum angle, plus the time t_m_ was used to represent the end of motion time (Tr) that would result if unimpeded by the participant. This is shown by the light grey construction lines on the angle profile in Figure 2 (top plot).

An acceleration profile was then constructed between T1 and Tr to represent a constant jerk curve as shown in Figure 2 (bottom plot, dashed-line). Because the peak acceleration is quite sensitive to the range of movement and duration, the acceleration curve was arbitrarily given a peaks of +/−1000 deg/s, then integrated to estimate the velocity profile, and integrated a second time to estimate the angle profile. Given the known range of the actual profile, the angle curve was then scaled to fit the lower-to-upper limits of motion. Numerical differentiation (5-point Lagrangian method) was then used to compute velocity and acceleration profiles for the reference motion and actual motion.

Figure 2 shows examples of model derivation for a participant with a normal kinematic profile during passive stretch and for a participant with a spastic response during passive stretch. As can be seen, the assumption of constant jerk represented quite accurately the kinematic profile in a participant with normal tone (Figure 2a). In contrast, the kinematic departure from the reference profile was clearly evident for the participant with moderate spasticity (Figure 2b).

#### Kinematic model of spastic onset

A sudden resistive force to a passive motion caused by muscle stretch-reflex will be reflected by a sudden reduction in velocity rate of change (i.e. deceleration) of the imposed movement profile. Because the system already has inertia, the involuntary muscle activation of muscles during passive stretching should correspond to the peak acceleration departure from a “reference” kinematic profile that would have occurred if no resistance was encountered.

**Figure 3:**
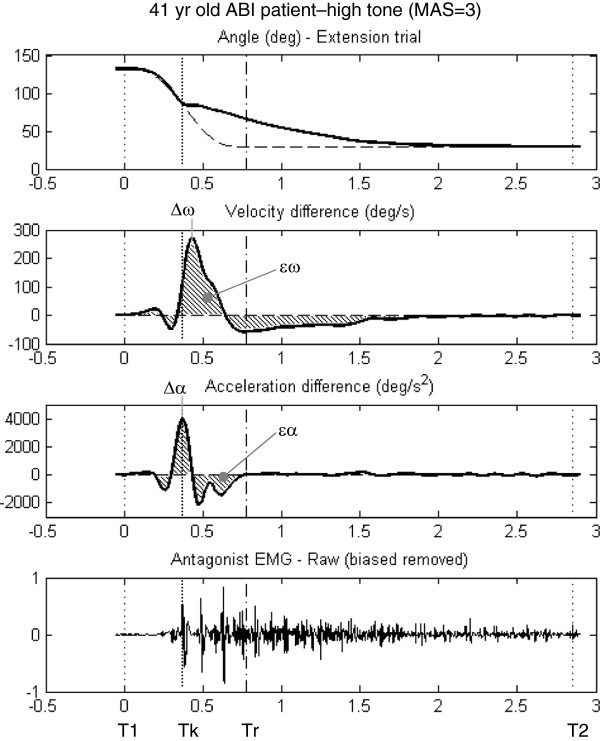
**Example of muscle onset prediction from kinematic data captured during stretch**-**reflex testing of a patient with high flexor tone (MAS = 3).** The top panel shows the patient’s angular displacement curves for actual and reference profiles. The next two panels show velocity (ω) and acceleration (α) departures from the reference profile. The peak departure acceleration preceding the peak departure velocity is taken to be the departure onset time (Tk). The kinematic peaks (Δω and Δα) were used to represent discrete measures of kinematic departure, and the area-under-the-curve (hatched region) was used to represent the density measures of kinematic departure (ϵω and ϵα).

Therefore, the time of absolute maximum departure in velocity from the reference profile during the movement was first determined (Δω), and then the time of peak acceleration (Δα) that occurred prior to this peak departure velocity was used as the onset time (Tk). An example is shown in Figure 3 for a participant with high spasticity during an elbow extension stretch-reflex test. Raw EMG signals are included to illustrate the correspondence of Tk to EMG onset as indicated by elbow flexor muscle’s activity.

This allowed three time windows to be defined for data processing: “Pre-onset” was defined by the time window between start of movement and spastic onset (T1-Tk), “Post-onset” was defined by the time window between spastic onset and end of the reference profile (Tk-Tr), and “Post-ref” was defined as the time window between Tr and when the motion trial actually completed (Tr-T2).

#### EMG signal processing

**Figure 4:**
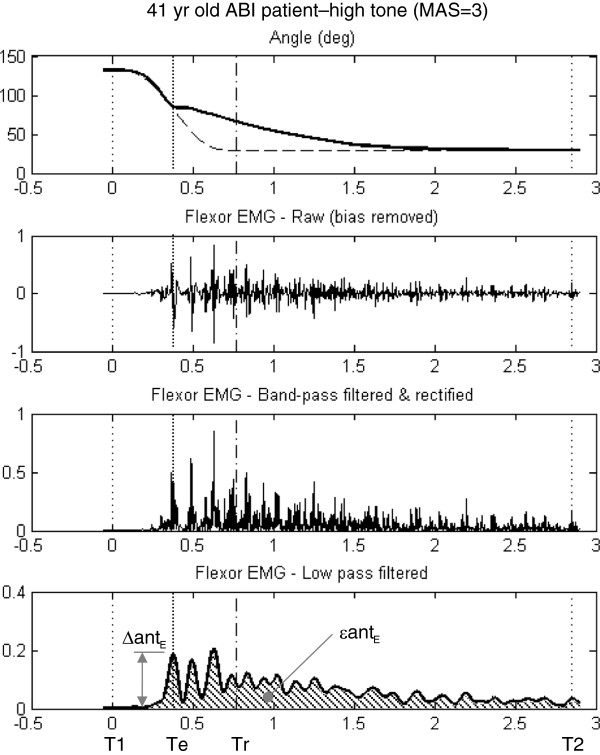
**Example of EMG processing and muscle onset prediction from EMG data stretch**-**reflex testing of a patient with high flexor tone (MAS = 3).** The top panel shows the patient’s angular displacement curves for actual and reference profiles. The next two panels show raw and rectified signals, respectively, and the bottom panel shows low-pass (10 Hz) filtered EMG waveform. Onset time (Te) was determined by the first peak that surpassed a 20% change in EMG amplitude from pre-onset EMG. The change in EMG amplitude was used to represent the discrete change in EMG intensity antagonist (Δant_E_) muscle, and area-under-the-curve was used to represent density measurement of EMG amplitude over the trial duration for antagonist (ϵant_E_) muscles (hatched region).

EMG data were processed by first band-pass filtering (20–400 Hz) the signal, followed by rectification, and then low-pass filtering at 10 Hz. A zero-lag, 4^th^ order Butterworth filter was used. Figure 4 shows the signal processing steps for a participant with high spasticity (as used for Figure 3). A major challenge in normalizing EMG in people with spasticity is that maximal voluntary isometric contractions (MVIC) can be very weak and non-representative of the signal intensity of the involuntary stretch-reflex. Therefore, we did not scale EMG signals to MVIC.

Also, it should be noted that in order to simplify the explanation, the muscle being stretched during a stretch-reflex test will be referred to as the antagonist muscle, while the muscle on the opposite side of the joint (not being stretched) will be referred to as the agonist. While it is understood that agonist/antagonist designations refer to voluntary muscle actions, we are merely using them as anatomical designations so the protocol can be described generically for flexion or extension stretch-reflex tests.

### Data analysis

#### Prediction of onset time

First, we determined how well the kinematic prediction of muscle onset (Tk) agreed with the muscle onset as indicated by the EMG signal. To do this the antagonist processed EMG signal was scaled to 0–1 by dividing the signal values by the maximum EMG value recorded during the trial. Then the scaled-EMG signal was averaged between start of movement time T1 and onset time Tk (pre-onset EMG, preE) and between Tk and end of motion Tr (post-onset EMG, posE) of the reference profile. If the average scaled-EMG signal increased by more than 20% (posE-preE > .2), the time of the first peak EMG signal (in the window T1-T3) was selected as EMG onset time (Te). If the change in signal was less than 20%, a score of “no-onset” was assigned to the trial. Furthermore, if the trigger velocity was below100 deg/s the trial was registered as “no-onset”. For trials with clear onsets, the difference in onset time δT was calculated from Te-Tk.

#### Quantifying muscle reflex intensity

Involuntary reflex response due to stretching is a discrete event; therefore variables extracted to represent muscle reflex intensity were also discrete events. These metrics represent “maximal” departures from reference. Two kinematic variables were selected: the peak magnitudes of departures from reference for velocity (Δω) and acceleration (Δα) (see Figure 3). Also, peak (non-scaled) EMG at time Te was selected. The mean EMG signal for pre-onset was subtracted from this value to give a measure of the change in EMG signal intensity. This was done for both antagonist (Δant_E_) and agonist (Δago_E_) muscle signals (see Figure 4).

#### Quantifying muscle reflex density

Prolonged resistance to passive motion is continuous, rather than a discrete event. Therefore, metrics extracted to represent resistance to passive motion were based on gross deviations from reference, rather than instantaneous deviations. These metrics represent “density” departures from reference (similar to what others refer to as muscle reflex gain [3]). For kinematic variables, we quantified the root mean square departure for angular velocity (ϵω) and acceleration (ϵα) between the start of motion and the actual end of motion (T1-T2) (see Figure 3). EMG departures from “reference” (no signal) were summarized by area under the curve over T1-T2 for antagonist (ϵant_E_) and agonist (ϵago_E_) muscles (see Figure 4).

#### Statistical analysis

##### Objective 1

This objective was to explore the kinematic and EMG data from the BioTone and develop a framework for extracting metrics relevant to assessing spastic hypertonia. Based on biomechanical principles and prior research on the neurophysiological response to passive stretching of spastic muscle, our hypotheses are as follows. Note the main hypothesis is stated with the null hypothesis requirement in parentheses:

H1.1: *Kinematic prediction of SR onset time* (Tk) *is equivalent to onset time* (Te) *indicated by EMG signal* (*or* δT = 0) (null hypothesis accepted).

H1.2: *Discrete kinematic variables* (Δω *and* Δα) *will correlate positively with corresponding antagonist EMG intensity* (Δant_E_) (null hypothesis rejected), *but will not correlate with agonist EMG intensity* (Δago_E_) (null hypothesis accepted); and

H1.3: *Density kinematic variables* (ϵω *and* Δα) *will correlate positively with corresponding antagonist EMG density* (ϵ*ant*_*E*_) (null hypothesis rejected), *but will not correlate with agonist EMG density* (ϵ*ago*_*E*_) (null hypothesis accepted).

Hypothesis H1.1 was tested using a one-sample *t*-test to determine if δT was different from zero, and hypotheses H1.2 and H1.3 were tested with 1-tailed correlation analyses. A one-tailed test is justified because we expect the correlation between biomechanical (kinematic) and electrophysiological (EMG) outcomes to be directly proportional.

##### Objective 2

This objective was to explore the above framework for establishing the construct validity of the BioTone measurement system for clinically meaningful assessment of muscle spasticity. Based on prior literature regarding the relationships between clinical and neuro-biomechanical measures of muscle spasticity, we hypothesized:

H2.1: *Discrete metrics will correlate positively with clinical MAS score*, *and negatively with strength and PROM* (null hypothesis rejected); and

H2.2: *Density metrics will correlate positively with clinical MAS score*, *and negatively with strength and PROM* (null hypothesis rejected);

Hypothesis H2.1 and H2.2 were tested with 1-tailed correlation analyses. Similarly there is a rationale for using a 1-tailed test given the expectation of directionality of these relationships.

Because MAS scores are ordinal in nature, and EMG signals are notoriously non-normally distributed, all correlations were performed using a Spearman rank correlation analysis, with α = .05 for statistical significance. Paired t-tests were used to compare onset time of muscle activation between kinematic and EMG measures. SPSS version 20 (IBM Corp.) was used for all statistical analyses.

## Results

Results of strength and ROM testing are shown in Table 1. Clearly the participants had a wide range of strengths for flexor (biceps) and extensor (triceps) muscles. Across the sample tested, the mean flexor force generating capacity was 20.2 N (SD = 17.1 N, Max = 47.1 N, Min = 3.72 N), and mean extensor force generating capacity was 30.3 N (SD = 19.9 N, Max = 68.8 N, Min = 6.16 N). Mean PROM was 112° (SD = 17.9°, Max = 131°, Min = 75.1°).

Kinematic and EMG metrics from the BioTone system and the clinical MAS score, for elbow extension and elbow flexion, are shown in Tables 3 and 4 respectively. In general, the sample studied had significantly (p = .030) more spasticity in elbow flexors (mean clinical MAS = 2.22+/−1.48, Max = 4, Min = 0) during elbow extension trials compared to elbow extensors (mean clinical MAS = 1.11+/−1.27, Max = 3, Min = 0) during elbow flexion trials. This was also consistent with greater mean values for all kinematic and EMG metrics for elbow extension trials compared to elbow flexion trials.

**Table 3:** Stretch-reflex test results for the study participants during elbow extension testing, stretching the biceps-bracii

	**Discrete measures**				**Density measures**				*Clinical
**ID**	Δ**ω**	Δ**α**	Δ**ant**_**E**_	Δ**ago**_**E**_	**ϵω**	**ϵα**	**ϵant**_**E**_	**ϵago**_**E**_	**MAS**
1	285.9	4113.	.2441	.0075	101.2	849.8	.1862	.0181	4 (3)
2	42.65	770.9	.0150	.0065	13.57	193.0	.0095	.0109	0 (0)
3	72.12	1205.	.1187	.0968	24.47	379.4	.0797	.0883	2 (1+)
4	155.9	1735.	.0995	.0335	52.61	539.0	.1337	.1422	4 (3)
5	168.4	3812.	.2434	.0128	40.58	846.6	.1435	.0190	2 (1+)
6	200.1	3294.	.1238	.0026	66.65	781.3	.1083	.0333	3 (2)
7	58.83	1528.	.0410	.0019	20.60	354.1	.0591	.0190	3 (2)
8	218.0	3035.	.0606	.0119	71.97	855.6	.0725	.0766	2 (1+)
9	72.53	1580.	.0665	.0386	18.58	401.2	.0360	.0237	0 (0)

Discrete measures include maximal kinematic and EMG deviations from reference at spastic onset. Density measures include area-under-the-curve measures of deviations from reference across the trial. Also included is the clinical MAS score recorded by the therapist.

^*^ Clinical score was assessed by therapist. We show the score used in the data analysis, with the recorded clinical score shown in parenthesis.

**Table 4:** Stretch-reflex test results for the study participants during elbow flexion testing, stretching the triceps-bracii

	**Discrete measures**				**Density measures**				*Clinical
**ID**	Δ**ω**	Δ**α**	Δ**ant**_**E**_	Δ**ago**_**E**_	**ϵω**	**ϵα**	**ϵant**_**E**_	**ϵago**_**E**_	**MAS**
1	138.5	1482.	.0538	.0066	58.76	499.5	.0642	.0343	3 (2)
2	44.68	660.0	.0029	.0015	19.68	248.1	.0074	.0171	0 (0)
3	36.50	666.7	.0442	.0227	12.41	207.6	.0212	.0541	0 (0)
4	59.62	569.2	.0737	.0730	23.61	268.4	.0470	.1059	1 (1)
5	169.6	2175.	.1223	.0412	65.76	639.0	.0683	.0836	2 (1+)
6	121.5	1576.	.0643	.1008	56.94	477.6	.0643	.0659	3 (2)
7	75.93	1473.	.0089	.0015	19.84	317.6	.0110	.0301	0 (0)
8	117.0	1917.	.0106	.0038	39.56	563.8	.0543	.0426	1 (1)
9	50.23	630.9	.0093	.0068	15.25	249.3	.0102	.0246	0 (0)

Discrete measures include maximal kinematic and EMG deviations from reference at spastic onset. Density measures include area-under-the-curve measures of deviations from reference across the trial. Also included is the clinical MAS score recorded by the therapist.

^*^ Clinical score was assessed by therapist. We show the score used in the data analysis, with the recorded clinical score shown in parenthesis.

### Objective #1: Relationships among kinematic and EMG data during elbow stretch-reflex

For hypothesis H1.1, the null hypothesis was accepted as anticipated. There was no significant difference between spastic onset time predicted from kinematic data and EMG data, for elbow extension trials (n = 7 onset trials, mean δT = −.017, 95% CI [−.087,.054], *p* = .575) or elbow flexion trials (n = 6 onset trials, mean δT = −.013, 95% CI [−.095,.069], *p* = .695).

For hypothesis H1.2, the null hypothesis was rejected as anticipated for elbow extension trials, but only partially so for elbow flexion trials. For elbow extension trials, there was a significant positive correlation for antagonist EMG increase (Δant_E_) with maximum velocity (Δω, *r* = .683, *p* = .021) and acceleration (Δα, *r* = .783, *p* = .006) departures from reference. For elbow flexion trials there was a borderline significant positive correlation for antagonist EMG increase (Δant_E_) with maximum velocity (Δω, *r* = .583, *p* = .500) departure, but no significant correlation with acceleration (Δα, *r* = .333, *p* = .190) departure. As hypothesized, the kinematic variables did not correlate significantly with agonist EMG increase (r < .22, p > .29) for both extension and flexion trials (null hypothesis accepted). These results are summarized in Table 5.

**Table 5:** **Spearman correlation coefficient (*r*) and significance (*p*) between kinematic variables and EMG intensity during stretch reflex (N = 9 subjects)**

**Kinematic metrics**		**Muscle EMG metrics**			
		**Extension test**^**†**^		**Flexion test**^**‡**^	
**Discrete**		**Δant**_**E**_	**Δago**_**E**_	**Δant**_**E**_	**Δago**_**E**_
Δω	*r*	**0.683**	0.000	**0.583**	0.217
	*p*	**0.021**	0.500	**0.050**	0.288
Δα	*r*	**0.783**	−0.100	0.333	0.033
	*p*	**0.006**	0.399	0.190	0.467
**Density**		**ϵant**_**E**_	**ϵago**_**E**_	**ϵant**_**E**_	**ϵago**_**E**_
ϵω	*r*	**0.767**	0.283	**0.867**	0.467
	*p*	**0.008**	0.230	**0.001**	0.103
ϵα	*r*	**0.667**	0.233	**0.800**	0.367
	*p*	**0.025**	0.273	**0.005**	0.166

Bold values significant at *p* ≤ .05.

† ant_E_ = biceps-bracii EMG intensity; ago_E_ = triceps-bracii EMG intensity.

‡ ant_E_ = triceps-bracii EMG intensity; ago_E_ = biceps-bracii EMG intensity.

For hypothesis H1.3, the null hypothesis was rejected as anticipated for both elbow extension and flexion trials. For elbow extension trials, there was a significant positive correlation for antagonist EMG density (ϵant_E_) with density of velocity (ϵω, *r* = .767, *p* = .008) and acceleration (ϵα, *r* = .667, *p* = .025) departures from reference. Likewise, for elbow flexion trials, there was a significant positive correlation for antagonist EMG density (ϵant_E_) with density of velocity (ϵω, *r* = .867, *p* = .001) and acceleration (ϵα, *r* = .800, *p* = .005) departures from reference. Also hypothesized, the kinematic variables did not correlate significantly with agonist EMG increase (*r* < .47, *p* > .10) for both extension and flexion trials (null hypothesis accepted). These results are summarized in Table 5.

Although not a planned hypothesis test, strong correlations were also found between the muscle reflex intensity (discrete) metrics and muscle reflex density metrics (*r* > .8, *p* < .01).

### Objective #2: Relationships among sensor and clinical data during elbow stretch-reflex

**Figure 5:**
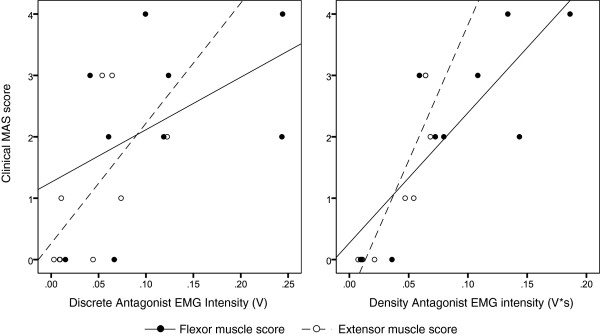
**Scatter plots showing the relationship between discrete and density measures of BioTone EMG and clinical MAS score for elbow extension (flexor muscle) and elbow flexion** (**extensor muscle**) **trials.** Left: Correlation between discrete EMG responses and MAS score. Right: Correlation between density EMG responses and MAS score.

For hypothesis H2.1, the null hypothesis was not rejected (as anticipated) for elbow extension trials, and was partially rejected for elbow flexion trials. For elbow extension trials, the direction of correlations were correctly hypothesized, but correlation coefficients were non-significant between kinematic and EMG metrics and clinical MAS (r < .52, p > .07), muscle strength (r > −.57, p > .06) and PROM (r > −.28, p > .14). However, for elbow flexion trials clinical MAS correlated positively with maximal velocity departure (Δω, *r* = .843, *p* = .002) and antagonist EMG increase (Δant_E_, *r* = .738, *p* = .012). Elbow extensor strength also correlated negatively with EMG increase during spastic contraction (*r* = −.717, *p* = .015). These results are summarized in Tables 6 (extension test) and 7 (flexion test). Regression plots are shown in Figure 5 (left).

**Table 6:** **Spearman correlation coefficient (*r*) and significance ( *p*) between BioTone spasticity measures and clinical MAS during elbow extension (biceps-bracii) stretch-reflex, as well as strength and passive ROM measures**

**BioTone variables**		**MAS score**	**Strength (N)**		
		**Flexor muscle**	**Extensor**	**Flexor**	**PROM (**^**o**^**)**
Discrete					
Δω	*r*	0.481	−0.167	0.000	−0.267
	*p*	0.095	0.334	0.500	0.244
Δα	*r*	0.524	−0.350	−0.317	−0.283
	*p*	0.074	0.178	0.203	0.230
Δant_E_	*r*	0.481	−0.567	−0.467	0.133
	*p*	0.095	0.056	0.103	0.366
Δago_E_	*r*	−0.266	−0.133	0.167	0.417
	*p*	0.244	0.366	0.334	0.132
Density					
ϵω	*r*	**0.687**	−0.333	−0.100	−0.333
	*p*	**0.020**	0.190	0.399	0.190
ϵα	*r*	0.361	−0.150	0.017	−0.317
	*p*	0.170	0.350	0.483	0.203
ϵant_E_	*r*	**0.721**	**−0.783**	**−0.600**	−0.067
	*p*	**0.014**	**0.006**	**0.044**	0.432
ϵago_E_	*r*	0.206	−0.183	0.217	−0.283
	*p*	0.297	0.318	0.288	0.230

Bold values significant at *p* ≤ .05.

**Table 7:** **Spearman correlation coefficient (*r*) and significance (*p*) between BioTone spasticity measures and clinical MAS during elbow flexion (triceps-bracii) stretch-reflex, as well as strength and passive ROM measures**

**BioTone variables**		**MAS score**	**Strength (N)**		
		**Flexor muscle**	**Extensor**	**Flexor**	**PROM (**^**o**^**)**
Discrete					
Δω	*r*	**0.843**	−0.317	−0.450	−0.433
	*p*	**0.002**	0.203	0.112	0.122
Δα	*r*	0.580	−0.033	−0.217	−0.350
	*p*	0.051	0.466	0.288	0.178
Δant_E_	*r*	**0.738**	**−0.717**	−0.467	−0.133
	*p*	**0.012**	**0.015**	0.103	0.366
Δago_E_	*r*	0.527	−0.417	−0.117	0.000
	*p*	0.072	0.133	0.383	0.500
Density					
ϵω	*r*	**0.896**	−0.417	−0.433	−0.333
	*p*	**0.001**	0.132	0.122	0.190
ϵα	*r*	**0.738**	−0.200	−0.300	−0.533
	*p*	**0.012**	0.303	0.216	0.070
ϵant_E_	*r*	**0.896**	−0.483	−0.400	−0.317
	*p*	**0.001**	0.094	0.143	0.203
ϵago_E_	*r*	0.553	**−0.700**	−0.383	−0.300
	*p*	0.061	**0.018**	0.154	0.216

Bold values significant at *p* ≤ .05.

For hypothesis H2.2, null hypothesis was partially rejected for both elbow extension and flexion trials. For elbow extension trials, clinical MAS correlated positively with density of velocity departure (ϵω, *r* = .687, *p* = .020) and antagonist EMG density (ϵant_E_, *r* = .721, *p* = .014). Furthermore, antagonist EMG density was negatively correlated with both extensor (*r* = −.783, *p* = .006) and flexor (*r* = −.600, *p* = .044) muscle strength. For elbow flexion trials, clinical MAS correlated positively with density of both velocity (ϵω, *r* = .896, *p* = .001) and acceleration departure (ϵα, *r* = .738, *p* = .012), and antagonist EMG density (ϵant_E_, *r* = .896, *p* = .001). Interestingly, agonist EMG density was negatively correlated with strength extensor strength (*r* = −.700, *p* = .018). These results are summarized in Tables 6 (extension test) and 7 (flexion test). Regression plots are shown in Figure 5 (right).

## Discussion

### Why is it important to objectively quantify muscle spasticity?

Ongoing management of problematic spasticity is commonly required in people with brain or spinal cord injury or disease [1]. Although the neurological basis of spasticity is relatively well documented [24], spasticity is not very well understood in terms of its clinical presentation [25], nor is there consensus on how best to clinically assess spasticity. Clinical rating scales, such as the MAS [6] are commonly used in both practice [26] and in clinical trials [8], but the literature is unclear regarding their validity. While some laboratory-based studies suggest such scales are insufficient for measuring spasticity [11,12,16] others report the MAS correlates with neurophysiological response during passive stretching [18-21].

The cost of rehabilitation and management of problematic spasticity and related complications (pain, contracture, etc.) can be extraordinarily high [27] and “cost-benefit” research of pharmacological treatments often use standard clinical assessment tools such as the MAS [28,29] as outcomes measurements. Furthermore, according to Pandyan et al. [8] all randomized clinical trials on stroke therapy published between October 1989 and October 2004 used the MAS as a primary outcome measure. Therefore, it is not only critical to determine what the MAS is actually measuring, but there is a clear need to explore better ways to incorporate more objective assessment of spasticity into clinical research and practice. Indeed, there is a growing interest in this topic [8,14,15], but there is little evidence of clinical uptake of these concepts.

### What are the barriers and enablers to clinical adoption of technology?

One significant barrier to the clinical adoption of technologies that can acquire objective measures of spasticity is that the vast majority of these proposed technologies are not well suited to a clinical environment. For example, several studies have developed sophisticated measurement and modeling approaches to better characterize spasticity [10-12,16,18,21,30]. These approaches require specialized motor driven mechanical systems that are not only cost and space prohibitive in a rehabilitation clinic, but require significant training and ongoing technical maintenance. Isokinetic dynamometry systems have also shown promise for spasticity assessment [15,31], and may in general be more accessible than specialized systems, but still cannot be widely adopted as only a small percentage of rehabilitation clinics are equipped with this technology. Although these studies have contributed greatly to our understanding of biomechanical and neurophysiological aspects of spasticity, the proposed solutions are unlikely to be widely adopted by clinics for routine use.

Given the constraints of a clinical environment, technology solutions for spasticity assessment must be portable, inexpensive, require minimal maintenance and technical training, and most importantly must be clinically valid and efficient to use. The only class of technology that fits these criteria is wearable sensor technology. Wearable technologies for sensing kinematics and muscle activity can deliver quantitative objective information that is of interest to the treating therapist. Wearable technologies are small, lightweight and generally unobtrusive, and could allow the clinician to perform unencumbered routine physical examinations while monitoring and collecting important clinical variables.

For example, Pandyan et al. [8,13] have reported on a wearable system that included an electrogoniometer, 2-channel EMG system, and load cell apparatus attached to the participant’s forearm. Motion was applied by the tester to the forearm apparatus to measure therapist driving force during stretch-reflex testing, and combined with the kinematic data to assess resistance to passive motion and correspondence to EMG activity. While this provides a solution for objective stretch-reflex assessment, the forearm load cell device proposed by Pandyan and colleagues may not be suitable for routine testing in a clinical setting for a number of reasons, most critically, the clinician is moving the forearm apparatus and not the forearm directly, which changes the clinical testing protocol and introduces unknown artefact due to motion at the interface of apparatus and participant forearm. The analysis approach we propose shows that by using a kinematic model and measurement system, an estimate of the spastic muscle interference force (acceleration disturbance) can be obtained without an external force transducer device, which correlates strongly with EMG intensity metrics.

The use of inertial sensor technology (accelerometers, gyroscopes, etc.) is becoming increasingly popular in rehabilitation research, and fits the criteria of being unobtrusive, inexpensive, relatively easy to use without substantial training, and requires little maintenance. An example of applying this technology for spasticity assessment is described by Paulis et al. [14]. Their study used inertial sensors on the forearm to evaluate the test-retest reliability of the Tardieu scale: a variant of the MAS that includes passive stretching at different velocities [7]. However, the wearable system proposed did not include EMG measurement. A recent study [32], however, suggests that inertial sensors (as used in the study by Paulis and colleagues) are susceptible to electromagnetic field artefacts from nearby metallic objects, such as a wheelchair. This could be a significant concern for testing individuals in a clinical environment where special seating arrangements may be required (quadriplegic CP or SCI, for example).

### Can a wearable sensor system be used to assess spasticity in a clinical environment?

Our study reports preliminary results of a wearable sensor system (the BioTone) designed for routine clinical assessment of elbow spasticity that was tested in a clinical environment using standard clinical protocols. In this paper we focus on developing a framework for evaluating this technology as a tool for quantifying elbow spasticity during stretch-reflex tests, and provide preliminary evidence of the construct validity of such a wearable sensor system.

#### Neuro-biomechanical validity of the BioTone system

Our preliminary results strongly suggest that a simple wearable system (fibre-optic goniometer and 2-channel EMG) and trial-specific kinematic model of passive joint kinematics shows promise for quantifying spastic hypertonia in a clinical setting. The kinematic model was able to predict stretch-reflex muscle onset to within 20 ms of the EMG onset time. It also enabled extraction of biomechanical metrics that for the most part correlated strongly and significantly with measures of antagonist (stretching) muscle activity during stretch-reflex testing, and that did not correlate significantly with agonist (shortening) muscle activity (which remained inactive). This suggests our kinematic model is a valid approach for characterizing elbow muscle spasticity.

We also derived two sets of metrics from the kinematic and EMG data to represent the spastic reflex intensity and the effects of muscle resistance to stretching. For the latter, we did not have a measure of force or torque to calculate joint stiffness; therefore we modeled this effect by evaluating the “density” of the kinematic disruption and EMG responses over the duration of the trial. Conversely, spastic reflex intensity was assessed using “discrete” measures of the kinematic disruption at onset and the corresponding intensity of muscle EMG at onset. Kinematic measures correlated well with EMG measures for both metrics, and were also highly correlated with one another, which is consistent with the parallel development of spastic reflex and resistance to passive motion that occurs with upper motor neuron syndrome [25].

#### Clinical validity of the BioTone system

Unfortunately there is no clinical “gold-standard” for assessing muscle spasticity, therefore we examined the relationship between BioTone measures of spastic hypertonia and the clinical MAS score. Other lab-based studies have explored relationships between neuro-biomechanical variables and the clinical MAS score, and most have reported moderate to low correlations [11,12,16,17]. However, these studies used motor driven systems to manipulate the joint, where MAS scoring was performed separate from instrumented measurements. In our study, the MAS measurement was acquired in the same manner as the instrumented passive stretch-reflex test. This might explain why the correlation between some BioTone spasticity metrics and clinical MAS were somewhat higher than previously reported in the literature. It is also interesting to note that other studies measuring EMG during administration of MAS scoring also report higher correlations [18,20]. This may suggest that lab-based assessment of spasticity that use different testing protocols (motor driven movements of the limb) cannot be used directly to validate or invalidate clinical assessment practices.

Finally, although there were few significant correlations, the relationship between the BioTone spasticity measures and strength (and PROM) was consistently negative, suggesting an inverse relationship between voluntary muscle strength and degree of spasticity. A larger sample will be required to determine if this apparent relationship can contribute to a better understanding of response to passive stretching in people with elbow spasticity.

#### Limitations

There are several limitations to the present study. Data collected from a small sample of participants recruited at the beginning of a large multi-site study were used. Future follow-up studies using data from this project should clarify many of the findings observed in the present study. Most notably, however, is that the present study was focused on clinical implementation of a wearable sensor system; hence the “gold standard” chosen for construct validation was the Modified Ashworth. Future studies will be required to compare BioTone spasticity features to features extracted from lab-based objective approaches, such as the approach described by Mullick et al. [17]. Finally, the present study focusses only on impairment level measures and does not address the issues of functional consequences of spasticity. Future studies can address this important problem but only if an objective spasticity metric can be validated using technology that is accessible to the rehab community.

## Conclusions

We conclude from this preliminary study that the BioTone system captures clinically relevant information when used during passive stretch-reflex testing in participants with elbow spasticity due to a range of central neurologic disorders. A sensor system consisting of a 1-DOF goniometer and 2-channel EMG worn during passive stretch-reflex tests, in combination with a simple passive model of joint kinematics derived from the participants’ trials, was found to be useful for extracting metrics that correlate strongly with neurophysiological responses during elbow extension and flexion stretch-reflex tests. BioTone measures also correlated positively with clinical MAS score which provides construct validity for the clinical stretch-reflex test, and warrants further investigation of BioTone measures against lab-based gold standard measures of spasticity.

## Competing interests

The authors declare that they have no competing interests.

## Authors’ contributions

CM is the overall project and University PI, and developed the modeling and analysis approach, and wrote the manuscript. AS is the project engineer in charge of instrumentation and data collection, and implementation of clinical training and testing protocols. MJ is the project therapist in charge of clinical testing, and co-developed the training and testing protocol. CO is the SCCR PI on the project and supervised the clinical site activities. All authors contributed to the protocol development and goals, and all authors read and approved the final manuscript.

## References

[B1] Dromerick AW Gelber DA, Jeffry DR Clinical features of spasticity and principles of treatment Clinical Evaluation and Management of Spasticity 2002 Totowa, NJ: Humana Press 13 26

[B2] Sheean G The pathophysiology of spasticity Eur J Neurol 2002 9 1 3 9; discussion 53–61 11918643 10.1046/j.1468-1331.2002.0090s1003.x

[B3] Katz RT Rymer WZ Spastic hypertonia: mechanisms and measurement Arch Phys Med Rehabil 1989 70 2 144 155 2644919

[B4] Logan LR Rehabilitation techniques to maximize spasticity management Top Stroke Rehabi 2011 18 3 203 211 10.1310/tsr1803-203 21642058

[B5] Sheean G McGuire JR Spastic hypertonia and movement disorders: pathophysiology, clinical presentation, and quantification PM R 2009 1 9 827 833 10.1016/j.pmrj.2009.08.002 19769916

[B6] Bohannon RW Smith MB Interrater reliability of a modified ashworth scale of muscle spasticity Phys Ther 1987 67 2 206 207 3809245 10.1093/ptj/67.2.206

[B7] Patrick E Ada L The tardieu scale differentiates contracture from spasticity whereas the ashworth scale is confounded by it Clin Rehabil 2006 20 2 173 182 10.1191/0269215506cr922oa 16541938

[B8] Pandyan AD Van Wijck FM Stark S Vuadens P Johnson GR Barnes MP The construct validity of a spasticity measurement device for clinical practice: An alternative to the ashworth scales Disabil Rehabil 2006 28 9 579 585 10.1080/09638280500242390 16690587

[B9] Pisano F Miscio G Del Conte C Pianca D Candeloro E Colombo R Quantitative measures of spasticity in post-stroke patients Clin Neurophysiol 2000 111 6 1015 1022 10.1016/S1388-2457(00)00289-3 10825708

[B10] Lindberg PG Gaverth J Islam M Fagergren A Borg J Forssberg H Validation of a new biomechanical model to measure muscle tone in spastic muscles Neurorehabil Neural Repair 2011 25 7 617 625 10.1177/1545968311403494 21490269

[B11] Kumar RT Pandyan AD Sharma AK Biomechanical measurement of post-stroke spasticity Age Ageing 2006 35 4 371 375 10.1093/ageing/afj084 16675479

[B12] Alibiglou L Rymer WZ Harvey RL Mirbagheri MM The relation between ashworth scores and neuromechanical measurements of spasticity following stroke J Neuroeng Rehabil 2008 5 18 10.1186/1743-0003-5-18 18627628 PMC2515334

[B13] Pandyan AD Price CI Rodgers H Barnes MP Johnson GR Biomechanical examination of a commonly used measure of spasticity Clin Biomech (Bristol, Avon) 2001 16 10 859 865 10.1016/S0268-0033(01)00084-5 11733123

[B14] Paulis WD Horemans HL Brouwer BS Stam HJ Excellent test-retest and inter-rater reliability for tardieu scale measurements with inertial sensors in elbow flexors of stroke patients Gait Posture 2011 33 2 185 189 10.1016/j.gaitpost.2010.10.094 21131203

[B15] Damiano DL Quinlivan JM Owen BF Payne P Nelson KC Abel MF What does the ashworth scale really measure and are instrumented measures more valid and precise? Dev Med Child Neurol 2002 44 2 112 118 10.1017/S0012162201001761 11848107

[B16] Jobin A Levin MF Regulation of stretch reflex threshold in elbow flexors in children with cerebral palsy: a new measure of spasticity Dev Med Child Neurol 2000 42 8 531 540 10.1017/S0012162200001018 10981931

[B17] Mullick AA Musampa NK Feldman AG Levin MF Stretch reflex spatial threshold measure discriminates between spasticity and rigidity Clin Neurophysiol 2013 124 4 740 751 10.1016/j.clinph.2012.10.008 23146713

[B18] Cooper A Musa IM van Deursen R Wiles CM Electromyography characterization of stretch responses in hemiparetic stroke patients and their relationship with the modified ashworth scale Clin Rehabil 2005 19 7 760 766 10.1191/0269215505cr888oa 16250195

[B19] Bakheit AM Maynard VA Curnow J Hudson N Kodapala S The relation between ashworth scale scores and the excitability of the alpha motor neurones in patients with post-stroke muscle spasticity J Neurol Neurosurg Psychiatry 2003 74 5 646 648 10.1136/jnnp.74.5.646 12700310 PMC1738448

[B20] Skold C Harms-Ringdahl K Hultling C Levi R Seiger A Simultaneous ashworth measurements and electromyographic recordings in tetraplegic patients Arch Phys Med Rehabil 1998 79 8 959 965 10.1016/S0003-9993(98)90095-8 9710170

[B21] de Vlugt E de Groot JH Schenkeveld KE Arendzen JH van der Helm FC Meskers CG The relation between neuromechanical parameters and ashworth score in stroke patients J Neuroeng Rehabil 2010 7 35 10.1186/1743-0003-7-35 20663189 PMC2927906

[B22] Ashworth B Preliminary trial of carisoprodol in multiple sclerosis Practitioner 1964 192 540 542 14143329

[B23] Landry JS Sexton A Hughes G McGibbon CA A wearable sensor system for quantifying isometric elbow and knee joint strength 2012 Halifax: 35th Canadian Medical and biological engineering society

[B24] Nielsen JB Crone C Hultborn H The spinal pathophysiology of spasticity–from a basic science point of view Acta Physiol (Oxf) 2007 189 2 171 180 10.1111/j.1748-1716.2006.01652.x 17250567

[B25] Ivanhoe CB Reistetter TA Spasticity: The misunderstood part of the upper motor neuron syndrome Am J Phys Med Rehabil 2004 83 10 Suppl S3 S9 15448572 10.1097/01.phm.0000141125.28611.3e

[B26] Hsieh JT Wolfe DL Miller WC Curt A SCIRE Research Team Spasticity outcome measures in spinal cord injury: Psychometric properties and clinical utility Spinal Cord 2008 46 2 86 95 10.1038/sj.sc.3102125 17909559

[B27] Lundstrom E Smits A Borg J Terent A Four-fold increase in direct costs of stroke survivors with spasticity compared with stroke survivors without spasticity: the first year after the event Stroke 2010 41 2 319 324 10.1161/STROKEAHA.109.558619 20044535

[B28] Bensmail D Ward AB Wissel J Motta F Saltuari L Lissens J Cost-effectiveness modeling of intrathecal baclofen therapy versus other interventions for disabling spasticity Neurorehabil Neural Repair 2009 23 6 546 552 10.1177/1545968308328724 19228818

[B29] Shaw L Rodgers H Price C van Wijck F Shackley P Steen N BoTULS: a multicentre randomised controlled trial to evaluate the clinical effectiveness and cost-effectiveness of treating upper limb spasticity due to stroke with botulinum toxin type A Health Technol Assess 2010 14 26 1 113, iii-iv 20515600 10.3310/hta14260

[B30] Naghdi S Ansari NN Mansouri K Asgari A Olyaei GR Kazemnejad A Neurophysiological examination of the modified modified ashworth scale (MMAS) in patients with wrist flexor spasticity after stroke Electromyogr Clin Neurophysiol 2008 48 1 35 41 18338533

[B31] Pierce SR Johnston TE Shewokis PA Lauer RT Examination of spasticity of the knee flexors and knee extensors using isokinetic dynamometry with electromyography and clinical scales in children with spinal cord injury J Spinal Cord Med 2008 31 2 208 214 18581670 10.1080/10790268.2008.11760714 PMC2565481

[B32] Kendell C Lemaire ED Effect of mobility devices on orientation sensors that contain magnetometers J Rehabil Res Dev 2009 46 7 957 962 10.1682/JRRD.2008.09.0132 20104418

